# Lossless filter for multiple repeats with bounded edit distance

**DOI:** 10.1186/1748-7188-4-3

**Published:** 2009-01-30

**Authors:** Pierre Peterlongo, Gustavo Akio Tominaga Sacomoto, Alair Pereira do Lago, Nadia Pisanti, Marie-France Sagot

**Affiliations:** 1 Équipe-projet Symbiose, IRISA/CNRS, Campus de Beaulieu, Rennes, France; 2 Curso Experimental de Ciências Moleculares da Universidade de São Paulo, Brazil; 3 Instituto de Matemática e Estatística da Universidade de São Paulo, Brazil; 4 Dipartimento di Informatica, Università di Pisa, Italy; 5 Équipe BAOBAB, Laboratoire de Biométrie et Biologie Evolutive (UMR 5558); CNRS; Univ. Lyon 1, Villeurbanne Cedex, France and Équipe-Projet BAMBOO, INRIA Rhône-Alpes, France; 6 King's College, London, UK

## Abstract

**Background:**

Identifying local similarity between two or more sequences, or identifying repeats occurring at least twice in a sequence, is an essential part in the analysis of biological sequences and of their phylogenetic relationship. Finding such fragments while allowing for a certain number of insertions, deletions, and substitutions, is however known to be a computationally expensive task, and consequently exact methods can usually not be applied in practice.

**Results:**

The filter TUIUIU that we introduce in this paper provides a possible solution to this problem. It can be used as a preprocessing step to any multiple alignment or repeats inference method, eliminating a possibly large fraction of the input that is guaranteed not to contain any approximate repeat. It consists in the verification of several strong necessary conditions that can be checked in a fast way. We implemented three versions of the filter. The first is simply a straightforward extension to the case of multiple sequences of an application of conditions already existing in the literature. The second uses a stronger condition which, as our results show, enable to filter sensibly more with negligible (if any) additional time. The third version uses an additional condition and pushes the sensibility of the filter even further with a non negligible additional time in many circumstances; our experiments show that it is particularly useful with large error rates. The latter version was applied as a preprocessing of a multiple alignment tool, obtaining an overall time (filter plus alignment) on average 63 and at best 530 times smaller than before (direct alignment), with in most cases a better quality alignment.

**Conclusion:**

To the best of our knowledge, TUIUIU is the first filter designed for multiple repeats and for dealing with error rates greater than 10% of the repeats length.

## Background

Repeats in genomes come under many forms, such as satellites that are approximate repeats of a pattern of up to a few hundred base pairs appearing in tandem (consecutively) along a genome, segmental duplications that are defined as the duplications of a DNA segment longer than 1 kb, and transposable elements that are sequences of DNA that can move to different positions within a genome in a process known as transposition, or retrotransposition if the element was first copied and the copy then moved. The last two are repeats dispersed along a genome. Most such repeats appear in intergenic regions and were for long believed to be "junk" DNA, that is DNA that has no specific function although the proportion of repeated segments in a genome can be huge. Transposable elements alone cover up to, for example, 45% of the human and 80% of the maize genomes. This view of repeats as "junk" is changing though.

It is believed that transposable elements for instance may have been co-opted by the vertebrate immune system as a means of producing antibody diversity. Transposable elements are also thought to participate in gene regulation. This role had been suggested in the early 1950s by the discoverer of transposable elements herself, Barbara McClintock (she called such elements "mobile"), but she gave up publishing data supporting this idea in view of the strong opposition she was meeting from the academic world. The idea however stubbornly resisted denial or indifference and was resurrected much later. The paper of Lowe *et al*. [1] is just one of the last arguments in favour of a possible role for transposable elements in gene regulation. Indeed, by doing a genome-wide survey of 10402 characterised transposable elements, the authors found that these are most often located in regions of the human genome that contain very few genes, and show a strong preference for residing closest to genes involved in development and transcription regulation.

The relation between satellites and recombination, and therefore between satellites and certain types of rearrangements, seems also clear. Less clear is the relation, direct or indirect, that satellites may have with gene regulation although it is increasingly more suspected that such exists, for instance mediated by the chromatin [2]. Indeed, satellites represent one of a number of features characterising the chromatin whose different levels of packaging help define whether genes are available for expression (in regions called the euchromatin), or generally silenced (in regions called the heterochromatin). Other types of repeats continue also to be discovered. Among the more recent ones are the so-called "pyknons" [3]. These are apparently non random patterns of repeated elements that have been found more frequently in the 3' UTR region of genes than in other parts of the human genome. Cross-genome comparisons have revealed that many of the pyknons identified in human have instances in the 3' UTRs of genes from other vertebrates and invertebrates where they also appear over-represented. Although it is unclear how pyknons might have arisen, it is thus possible that they are involved in a new form of gene regulation.

The quantity of DNA in repeated sequences, the frequency of the repeat (that is, the number of times a given sequence is present per genome), and its conservation, show great variability across species. Frequencies from 100 to 1,000,000 have been observed, and the quantities of DNA involved range from 15 to 80 percent of a whole genome. Families of repeated sequences exhibit a degree of similarity among their members varying from perfect matching to matching of only two-thirds of the nucleotides. All these characteristics, plus the fact that in order to identify such repeats, it is necessary to work with whole genomes, that is with very long "texts", makes the identification of repeated elements a very hard computational problem.

In this paper, we focus on the problem of finding long multiple repeats that may appear dispersed along one whole genome or chromosome, or are common to different genomes/chromosomes. More precisely, since we are working with very long texts, we focus on the problem of filtering one or more sequences prior to a full identification of the multiple repeats that it may contain. Informally put, the idea is to eliminate from the input sequence(s) as many regions as possible that are sure not to contain any repeats of the type and characteristics specified. In some cases, the filter may be efficient enough that it eliminates all regions except those precisely corresponding to the repeats.

In the last few years, there has been an increasing number of papers on the topic of filtering sequences prior to further processing them. The motivations are varied, and include pattern matching [4-8], performing a local [9] or a global alignment [10,11], identifying repeats [12] or obtaining a multiple alignment [13,14].

This trend has been motivated by the fact that the problem of aligning sequences has scaled up considerably with the increasing number of genomes, notably of eukaryotes, that are being entirely sequenced and annotated. We say that a filter is *lossless *if it guarantees not to discard any fragment that may be part of a repeat. Filters, lossless or not, have been devised for comparing one sequence with itself [12] or two sequences pairwise [5,6,9,13]. Most filters rest on the idea that sequences that are reasonably similar contain patterns that match exactly. This is our case also.

To the best of our knowledge, filters for multiple repeats that take a multiple alignment condition into consideration have been addressed only in [15,16]. However, the authors in [15,16] allowed only for substituted basepairs between the different copies of a repeat, not indels. The method used in [15,16] is as a consequence quite different from the one in this paper. That method was based on a formula designed to characterise multiple repetitions without insertions nor deletions, and adopted a novel data structure employed to check the associated property. In the current paper, the conditions used are especially designed for edit distance and would not apply to Hamming distance. We therefore propose in this paper a filter, called TUIUIU, that: 1. is specifically taylored for multiple repeats, and 2. allows for a bounded edit distance among the different copies of a repeat, that is for deleted or inserted basepairs besides substitutions.

Since we do not know any other work that is a filter for multiple repeats, in particular with the same type of outcomes, we do not consider other methods to compare directly with TUIUIU, but we try instead to reproduce as much as possible the filtering conditions used by other filtering approaches. In this sense, the closest method we compare TUIUIU to is SWIFT[6].

The weakest of the filtering conditions we use corresponds to the filter used by SWIFT[6] for different purposes. Indeed, SWIFT was not developed with the same application in mind as TUIUIU. In particular, SWIFT is not a filter for multiple repeats, but a blast-like tool where the seeds are similarity regions with an error rate typically of at most 5%. Using TUIUIU as in SWIFT for pairwise comparison, we were able to improve the filtering power of SWIFT by applying two new conditions. TUIUIU is also able to deal with larger error levels, as high as 12%–14%. This implies however that bigger running times are also unavoidable. TUIUIU may be applied for finding two kinds of repeats: either repeats occurring in different sequences (like SWIFT) or repeats having multiple occurrences in a single sequence (something SWIFT cannot do). In both cases, the minimum number of occurrences, their length, and the minimum similarity degree between any pair of them, are user defined parameters.

We tested TUIUIU on random synthetic sequences with planted (*L*, *d*, *r*)-repeats using a very wide range of parameters. We also tested it on three sets of real data, the bacterium *Neisseria meningitidis *strain *MC58*, the human chromosome 22, and the dataset used in [13] denoted by *CFTR *(for Cystic Fibrosis Transmembrane conductance Regulator), adopting a similarly wide range of parameter sets. We found that our first additional filtration condition clearly leads to better results with negligible extra time, for all kinds of data and almost all parameter sets, with respect to the conditions previously used in the literature. Moreover, we also found that our second additional filtration condition considerably improves the selectiveness, with some time overhead, and becomes clearly advantageous mostly for large error rates.

Our method may also be used to find anchors for global multiple aligners. We thus expect that our filter could serve as a preprocessing step to a local multiple alignment tool. To this purpose, TUIUIU was applied as a preprocessing step of a multiple alignment application, leading to an overall execution time (filter plus alignment) on average 63 and at best 530 times smaller than before (direct alignment) and also, in some cases, to a qualitative improvement of the alignment obtained.

The rest of the paper is organised as follows. In the next section, we introduce formal definitions and the filtering conditions used in TUIUIU. In Section "Description of the algorithm", we first present the general structure of the algorithm, and we then specify the differences between the two versions of the algorithm (application to a single sequence or to a set of sequences). In Section "Complexity analysis", we provide a complexity analysis of both versions of the algorithm. In Section "Results and Discussion", we detail the experimental results obtained on biological DNA sequences, comparing different algorithmic strategies for filtering, including strategies used in other tools like SWIFT[6].

## Methods

### Preliminary definitions

A *sequence *is a concatenation of zero or more symbols from an alphabet Σ. In this work, we consider a sequence *s *of length *n *and we adopt the term *word *to denote a contiguous segment of *s*. We also consider an integer *m *≥ 2 and a set of sequences *s*_1_, *s*_2_,...,*s*_*m *_and in this case the term word is applied to a contiguous segment of one of the sequences *s*_1_, *s*_2_,...,*s*_*m*_. The sequence *s *of length *n *on Σ is represented by *s*[0]*s*[1]...*s*[*n *- 1], where *s*[*i*] ∈ Σ for 0 ≤ *i *<*n*. We denote by *s*[*i*, *j*] the word *s*[*i*]*s*[*i *+ 1]...*s*[*j*] of *s*. In this case, we say that the word *w *= *s*[*i*, *j*] *occurs at position i in s *or that *w starts at position i in s*. We say that two words *w *= *s*[*i*, *j*] and *w' *= *s*[*i'*, *j'*] (corresponding to occurrences *i *and *i'*) *overlap *if the intersection of the intervals [*i*, *j*] and [*i'*, *j'*] is non-empty.

We define a *q-gram *as a word of length *q*. The length of a word *w *is denoted by |*w*|. We recall that the edit distance between two given words is defined as the minimum number of edit operations that transform one into the other, where the considered *edit operations *are: symbol deletion, insertion or substitution.

**Definition 1 **((*L*, *d*, *r*)**-repeat**) *Given a sequence s and integers L *> 0, 0 ≤ *d *<*L and r *≤ 2, *an *(*L*, *d*, *r*)-repeat *is a set of r words in s not necessarily distinct but occurring at distinct positions, having length in the range *[*L *- *d*, *L *+ *d*], *being pairwise non overlapping, and such that the edit distance between any pair of them is at most d*.

**Figure 1:**
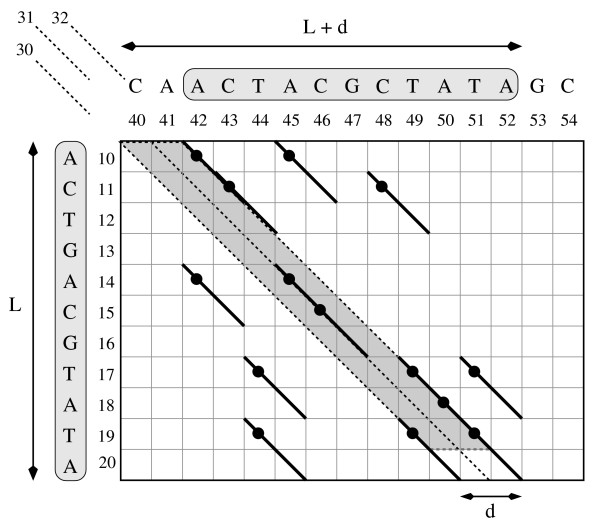
**A (*L*, *d*, 2)-repeat and a parallelogram**. An example a (*L*, *d*, 2)-repeat with *L *= 11, *d *= 2. Diagonals 30, 31, and 32 are shown. Among them, 30 and 32 have distance 2, while 30 and 31 (as well as 31 and 32) are consecutive. Assuming that *q *= 2, a *q*-hit is represented by a thicker diagonal of length 2 plus a small black circle representing its pair of coordinates. The *q*-hit (19, 49) refers to the *q*-gram *TA*, and 19 (resp. 49) is its first (resp. second) projection. The *q*-hit (17, 49) refers to the same *q*-gram *TA *but has a different first projection. The words inside the grey boxes are two distinct fragments of the same sequence *s*, namely *s*[10, 20] and s [42, 52]; they have length 11, and their edit distance is 2. We obtain one word from the other by deleting *s*[13] – hence no *q*-hits in positions 12, 13 – and by inserting s [48] – no *q*-hits in positions 47, 48. We have *p *= 6 and the set S of 7 *q*-hits in diagonals 31 and 32 satisfies the properties (1), (2), (3), (4) and (5). If we add the *q*-hit in diagonal 30 in order to obtain a new set $S^{\prime }$, properties (1), (2) still hold, but properties (3), (4) and (5) are no longer satisfied.

Figure 1 shows an example of an (*L*, *d*, 2)-repeat with *L *= 11 and *d *= 2.

Searching for multiple repeats means inferring all (*L*, *d*, *r*)-repeats of the input sequence(s), with parameters *L*, *d *and *r *given by the user. Since these are computationally hard to find, we propose a preprocessing step to mask out from the input sequences as many positions as possible that cannot belong to a word of length *L *that is part of an (*L*, *d*, *r*)-repeat. Since this condition is difficult to be quickly verified, we apply filtering conditions that are based on properties of the *q*-grams that simultaneously occur in two words of an (*L*, *d*, *r*)-repeat, as we shall see from now on. Some of the techniques presented here have being used since 1985 by Ukkonen [7] and many other authors, but we follow more closely the definitions, techniques and properties given by Rasmussen et al. [6].

**Definition 2 ***Given a sequence s, a q*-hit *h is defined as a pair *(*i*, *j*) *such that at positions i and j of s we have the same q-gram, that is s*[*i*, *i *+ *q *- 1] = *w *= *s*[*j*, *j *+ *q *- 1]. *We also say that the word w is the q*-gram of *h*. *For any pair h *= (*i*, *j*), *i *(*resp*. *j*) *is *the first projection (*resp*. second projection) *of h*.

**Definition 3 ***Given a q-hit h *= (*i*, *j*), *we say that the *diagonal of *h is *diag(*h*) = {*h' *= (*i'*, *j'*)|*j' *- *i' *= *j *- *i*}, *the set of all possible pairs of positions h' *= (*i'*, *j'*) *with the same difference of projections j *- *i*. *For convenience, we also say that this is *the diagonal *j *- *i *(*note that this number may be negative*). *We define the *difference of the diagonals of *h *= (*i*, *j*) and of *h' *= (*i'*, *j'*), *in this order*, *to be the difference *(*j *- *i*) - (*j'*- *i'*). *We say that two diagonals are *consecutive *if their difference is *1 *or *-1.

Figure 1 shows an example of diagonals and *q*-hits.

Let us consider a word *w *= *s*[*a*, *a *+ *L *- 1] of length *L *and another word *w' *= *s*[*a'*, *a' *+ *L *- 1]. One can notice that if we had an edit distance 0 between *w *and *w'*, then all the *L *- *q *+ 1 pairs (*a*, *a'*),...,(*a *+ *L *- *q*, *a' *+ *L *- *q*) would be *q*-hits (there could be more if the same *q*-gram occurs at more than one position of *s*). Roughly speaking, notice that any edit operation applied to one of the sequences will shift the diagonal of a *q*-hit by at most 1 and possibly remove at most *q q*-hits. Hence, if *w *and *w' *are distant by at most *d *edit operations, then there must be at least *p *= (*L *- *q *+ 1) - *qd q*-hits. The TUIUIU filter verifies this property (first introduced in the proof of Theorem 5.1 of [7]), that is:

(1) $$\text{there exists for }w\text{ and }w^{\prime }\text{ a set }S\text{ of }q\text{-hits of size at least }p=(L-q+1)-qd.$$

Moreover, in order to make the filtering condition more stringent, we also require that the set above is such that for any pair of *q*-hits *h *= (*i*, *j*) and *h' *= (*i'*, *j'*) in S, the following properties hold:

(2) |diag(*h*) - diag(*h'*)| ≤ *d*

(3) *i *≠ *i'*

(4) *j *≠ *j'*

(5) *i *<*i' *if and only if *j *<*j'*

In Figure 1 we can see examples for these properties that we motivate as follows. As mentioned above, an edit operation shifts the diagonal of a *q*-hit by at most one position. Thus, *d *edit operations can shift this diagonal by at most *d *positions, which explains property 2 (already used by the filter in [6]). We now prove that properties 3, 4 and 5 are also necessary conditions. These, to the best of our knowledge, were not used in previous filters while they will be considered in TUIUIU.

**Theorem 1 ***if w *= *s *[*a*, *a *+ *L *- 1] *and w' *= *s *[*a'*, *a' *+ *L *- 1] *are distant by at most d edit operations, then there are at least p *= (*L *- *q *+ 1) - *qd q-hits that pairwise verify properties 3, 4 and 5*.

*Proof*. Let *W *and *W' *be sequences on the alphabet Σ ∪ {-} where '-' ∉ Σ such that:

• there is no *i *∈ [0, |*W*| - 1] with both *W*[*i*] and *W'*[*i*] equal to '-';

• *L *≤ |*W*| = |*W'*| ≤ *L *+ *d*;

• the sequence obtained from *W *(resp. *W'*) by deleting all characters '-' is equal to *w *(resp. *w'*);

• *W *and *W' *are a representation of an optimal alignment between *w *and *w' *where the symbol '-' represents a gap and with cost function corresponding to the edit distance (0 for a match, 1 for any other edit operation).

Let now *X *be a sequence over the alphabet {M, D} such that:

• |*X*| = |*W*| = |*W'*|;

for all *i *from 1 to |*X*|, *X*[*i*] = *M *if *W*[*i*] = *W'*[*i*], else *X*[*i*] = *D*.

We now prove the following lemma.

**Lemma 1 ***There are at least p *= (*L *- *q *+ 1) - *qd distinct positions i such that for all j *∈ [0, *q *- 1], *X *[*i *+ *j*] = *M*, *that is, at least p *= (*L *- *q *+ 1) - *qd positions i where a run of Ms of size at least q begins*.

*Proof*. Obviously, there can be at most |*X*| - *q *+ 1 runs of *M*s of size at least *q *in *X*. Furthermore, each character *D *in *X *destroys at most *qd *runs of *M*s since there can be at most *d D*s in *X*. If *N *is the number of runs of *M*s of size at least *q *in *X*, we thus have:

*N *≥ (|*X*| - *q *+ 1) - *qd *≥ (*L *- *q *+ 1) - *qd*

runs of *M*s of size at least *q *in *X*.   □

By the way *X *was built and from Lemma 1, there are at least (*L *- *q *+ 1) - *qd *runs of *q M*s. Each pair of such runs corresponds to two *q*-hits themselves corresponding to two distinct *q*-grams in *w *(at positions *i *and *i'*) and in *w' *(at positions *j *and *j'*), proving conditions 3 and 4. Obviously, if the *q*-hit (*i*, *j*) (resp. (*i'*, *j'*)) occurs first, then *i *> *i' *and *j *> *j' *(resp. *i' *> *i *and *j' *> *j*), proving condition 5.   □

Observe that the above proof follows a reasoning somewhat similar to the one in [7]. In the remaining of this section, we introduce some terminology that we use to explain the actual steps performed by TUIUIU in order to verify the properties listed above.

For any word *w *= *s*[*a*, *a *+ *L *- 1], we want to check whether it belongs to an (*L*, *d*, *r*)-repeat. Suppose this is the case, that is, there exists words *w*_*k*_, for *k *= 1, 2,...,*r *- 1 such that *w *and *w*_*k *_have edit distance no more than *d*. For each pair of words *w *and *w*_*k*_, the computation of the edit distance would take as much as *θ*(*dL*) time for the best algorithm. Instead, we count the *q*-hits of these two words and we verify whether they are at least *p*, because there must be for *w *and *w*_*k *_a set of *q*-hits S that satisfies property (1). The *q*-hits could theoretically be as many as (*L *- *q *+ 1) × (*L *+ *d *- *q *+ 1). Nevertheless, if we also consider that property (2) must be satisfied by any pair of *q*-hits in S, then we can count *q*-hits only within the limited region of *d *+ 1 consecutive diagonals (like the diagonals 30, 31 and 32 in Figure 1) which includes no more than (*d *+ 1) × (*L *- *q *+ 1) possible *q*-hits. This shows us the convenience of sorting the *q*-hits by diagonals. Let us formalise this idea by introducing the notion of a *parallelogram*, that is found in [6].

**Definition 4 (parallelogram) ***Given a word w *= *s*[*a*, *a *+ *L *- 1] *of length L, and the set of d *+ 1 *consecutive diagonals *[*c*, *c *+ *d*], *with d *<*L*, *we define the respective parallelogram as the set of all pairs*:

Parall(*a*, *L*, *c*, *d*) = {(*i*, *j*)|*i *∈ [*a*, *a *+ *L *- *q*], *j *- *i *∈ [*c*, *c *+ *d*]}.

In Figure 1, the grey highlighted parallelogram represents the parallelogram Parall(10, 11, 30, 2). Notice that the *q*-hits (19, 49) and (19, 51) (are pairs that) *do *belong to Parall(10, 11, 30, 2), according to the definition.

A few observations can be made. The first is that *a *and *c *are such that the top left position of the parallelogram is (*a*, *a *+ *c*). Indeed, *a *and *a *+ *c *are the starting positions of the two words delimiting the parallelogram that contains the *q*-hits. Second, *a *+ *L *- *q *is the greatest position *i *such that the *q*-gram *s*[*i*, *i *+ *q *- 1] is a word of *w *= *s *[*a*, *a *+ *L *- 1]. The third observation is that the parallelogram Parall(*a*, *L*, *c*, *d*) has (*L *- *q *+ 1) × (*d *+ 1) pairs and this is its *size*. Finally, a pair *h *= (*i*, *j*) ∈ Parall(*a*, *L*, *c*, *d*) may or may not be a *q*-hit, depending on whether or not *w *[*i*, *i *+ *q *- 1] = *w *[*j*, *j *+ *q *- 1].

Given a word *w *= *s*[*a*, *a *+ *L *- 1], the parallelogram Parall(*a*, *L*, *c*, *d*) is used to check properties (1) and (2) for *w *against a word *w*_*k *_that is candidate to be one of the *r *- 1 words which, together with *w*, are part of an (*L*, *d*, *r*)-repeat. This is done in the following way. Let *w *and *w*_*k *_= *s*[*u*, *v*] be an (*L*, *d*, 2)-repeat, and consider an optimal alignment of these two words. The pairs of matched positions described in this alignment belong to no more than *d *+ 1 consecutive diagonals. In particular, there is a diagonal *c *with *c *such that *u *∈ [*c *+ *a*, *c *+ *d *+ *a*] and *v *∈ [*c *+ *a *+ *L *- 1, *c *+ *d *+ *a *+ *L *- 1] where the matched positions belong to the union of the diagonals *c*, *c *+ 1,...,*c *+ *d*. In this case, we say that the parallelogram Parall(*a*, *L*, *c*, *d*) *detects *this (*L*, *d*, 2)-repeat. This is why we can limit the search of the *q*-hits of *w *and *w*_*k *_to within the parallelogram.

**Figure 2:**
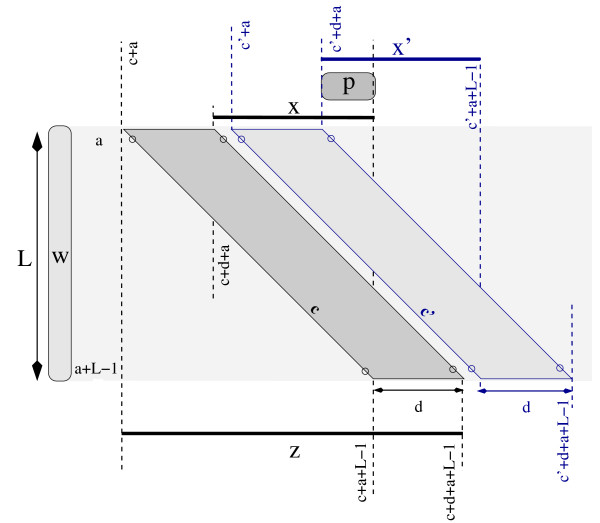
**Detection of (*L*, *d*, *r*)-repeats and two overlapping parallelograms**. Two parallelograms that overlap. The dark grey parallelogram in the figure detects *q*-hits between *w *= *s*[*a*, *a *+ *L *- 1] and any word *w*_*k *_= *s*[*i*, *j*] with *i *∈ [*c *+ *a*, *c *+ *d *+ *a*] and *j *∈ [*c *+ *a *+ *L *- 1, *c *+ *d *+ *a *+ *L *- 1]. The word *z *= *s*[*c *+ *a*, *c *+ *d *+ *a *+ *L *- 1] of length *L *+ *d *contains the word *w*_*k *_which in turn contains the word *x *= *s*[*c *+ *d *+ *a*, *c *+ *a *+ *L *- 1] of length *L *- *d*. Analogously for the light gray parallelogram, the word *z' *= *s*[*c' *+ *a*, *c' *+ *d *+ *a *+ *L *- 1] of length *L *+ *d *contains a word ${w^{\prime }}_{k}$ which in turn contains the word *x' *= *s*[*c' *+ *d *+ *a*, *c' *+ *a *+ *L *- 1] of length *L *- *d*. The words *w*_*k *_and ${w^{\prime }}_{k}$ are not shown because their length is variable. They necessarily overlap because they both contain the word *p *= *s*[*c' *+ *d *+ *a*, *c *+ *a *+ *L *- 1], that is the overlap between *x *and *x'*.

Consider now the word *x *= *s*[*c *+ *d *+ *a*, *c *+ *a *+ *L *- 1] of length *L *- *d *that is contained in *w*_*k*_, which in turn is contained in the word *z *= *s*[*c *+ *a*, *c *+ *d *+ *a *+ *L *- 1], having length *L *+ *d*. Both *x *and *z *are shown in Figure 2 for the darkest of the two parallelograms.

Notice that, since *d *<*L*, *x *is well defined because *c *+ *d *+ *a *≤ *c *+ *a *+ *L *- 1. Therefore, we have that any other (*L*, *d*, 2)-repeat {*w*, ${w^{\prime }}_{k}$} detected by the same parallelogram would be such that *w*_*k *_and ${w^{\prime }}_{k}$ overlap because they both contain *x*. This ensures that, for a word *w*, no two non-overlapping repeats could be detected by the same parallelogram.

We say that two parallelograms Parall(*a*, *L*, *c*, *d*) and Parall(*a*, *L*, *c'*, *d*) *overlap *if the words *x *= *s*[*c *+ *d *+ *a*, *c *+ *a *+ *L *- 1] and *x' *= *s*[*c' *+ *d *+ *a*, *c' *+ *a *+ *L *- 1] overlap. If *c *<*c'*, this happens if *c *+ *a *+ *L *- 1 ≥ *c' *+ *d *+ *a*. In other words, parallelograms Parall(*a*, *L*, *c*, *d*) and Parall(*a*, *L*, *c'*, *d*) overlap if and only if

|*c' *- *c*| <*L *- *d*.

In Figure 2 we can see two parallelograms that overlap, where the overlap *p *= *s*[*c' *+ *d *+ *a*, *c *+ *a *+ *L *- 1] between *x *and *x' *is highlighted.

In general, if Parall(*a*, *L*, *c*, *d*) detects the (*L*, *d*, 2)-repeat *w *= *s*[*a*, *a *+ *L *- 1] and *w*_*k *_= *s*[*u*, *v*], and Parall(*a*, *L*, *c'*, *d*) detects the (*L*, *d*, 2)-repeat *w *= *s*[*a*, *a *+ *L *- 1] and ${w^{\prime }}_{k}$ = *s*[*u'*, *v'*], and if the two parallelograms overlap, then the words *w*_*k *_= *s*[*u*, *v*] and ${w^{\prime }}_{k}$ = *s*[*u'*, *v'*] also overlap. We say that a set of parallelograms is *non-overlapping *if no two of them overlap.

Since *w *= *s*[*a*, *a *+ *L *- 1] and *w*_*k *_= *s*[*u*, *u *+ *L' *- 1] are two words with edit distance no more than *d*, the existence of a set of *q*-hits S that satisfies properties (1) and (2) implies that there are at least *p q*-hits inside a parallelogram Parall(*a*, *L*, *c*, *d*) with *c *such that *a *+ *c *≤ *u *≤ *a *+ *c *+ *d*.

We say that a parallelogram is *fine *if there are at least *p q*-hits inside the parallelogram. For example, the parallelogram highlighted in Figure 1 is fine, with a set $S^{\prime }$ of 8 *q*-hits inside. This leads us to our first filtering condition, that is easy to be efficiently checked:

*for any word w *= *s*[*a*, *a *+ *L *- 1], *we keep the positions in the interval *[*a*, *a *+ *L *- 1] *if there exist at least r *fine *non-overlapping parallelograms *Parall(*a*, *L*, *c*_*i*_, *d*), *with c*_*i *_∈ {*c*_1_,...,*c*_*r*_}.

It is worth noticing that *w *itself generates a fine parallelogram, which explains why we check the existence of *r fine *non-overlapping parallelograms instead of *r *- 1.

We are now going to see two more stringent filtering conditions leading to the additional conditions actually applied by TUIUIU.

First, we require that the set of *q*-hits inside the parallelogram satisfy property (3). This property simply ensures that two distinct *q*-hits of S do not share a first projection. We say that a parallelogram is *good *if and only if there are at least *p q*-hits inside the parallelogram such that no two of these *q*-hits have the same first projection. In Figure 1, the set S contains 7 *q*-hits that pairwise satisfy property (3), and hence the highlighted parallelogram is good. This leads us to our second filtering condition that is also easy to be efficiently checked:

*for any word w *= *s*[*a*, *a *+ *L *- 1], *we keep the positions in the interval *[*a*, *a *+ *L *- 1] *if there exist at least r *good *non-overlapping parallelograms *Parall(*a*, *L*, *c*_*i*_, *d*), *with c*_*i *_∈ {*c*_1_,...,*c*_*r*_}.

Second, we can further require that the set of *q*-hits S inside Parall(*a*, *L*, *c*, *d*) satisfies also property (5). We say that a good parallelogram is *excellent *if and only if there are at least *p q*-hits inside the parallelogram such that any two of them satisfy property (5). Given that S contains distinct *q*-hits, we have that, if property (5) holds for all pairs of *q*-hits in S, then properties (3) and (4) also do. Therefore, requiring property (5) for S guarantees that properties (3) and (4) hold as well. In Figure 1, the set S of 7 *q*-hits satisfies property (5) and, indeed, also satisfies property (3) and property (4). In fact, the highlighted parallelogram is excellent. This leads us to our third and last filtering condition that can be expected to be efficiently checked for general cases:

*for any word w *= *s*[*a*, *a *+ *L *- 1], *we keep the positions in the interval *[*a*, *a *+ *L *- 1] *if there exist at least r *excellent *non-overlapping parallelograms *Parall(*a*, *L*, *c*_*i*_, *d*), *with c*_*i *_∈ {*c*_1_,...,*c*_*r*_}.

### Necessary condition applied by TUIUIU

Given a sequence *s *and the parameters *L*, *d*, *r *described above, TUIUIU tries to keep only those positions of *s *inside an interval [*a*, *a *+ *L *- 1] such that the word *w *= *s*[*a*, *a *+ *L *- 1] of length *L *belongs to an (*L*, *d*, *r*)-repeat. Since this condition is hard to be efficiently verified, only necessary conditions are checked:

*for any interval *[*a*, *a *+ *L *- 1], TUIUIU* keeps these positions if there exists at least r *excellent (*or *fine *or *good *if the user so prefers*) *non-overlapping parallelograms *Parall(*a*, *L*, *c*_*i*_, *d*), *with c*_*i *_∈ {*c*_1_,...,*c*_*r*_}.

### Description of the algorithm

We now give an overview of the algorithm applied by TUIUIU whose pseudocode is provided in Appendix 1.

For any possible *q*-gram, we build the list of all its occurrences in *s*. The sum of the sizes of the |Σ|^*q *^occurrences lists is *n *- *q *+ 1. They are concatenated and stored in an array of *n *- *q *+ 1 positions and are accessed through |Σ|^*q *^pointers, one for each possible *q*-gram (line 1).

We move a sliding window *w *= *s*[*i*, *i *+ *L *- 1] of length *L *along *s *and only *q*-hits relative to this sliding window are considered. For each position *i*, we have to consider all possible parallelograms, i.e. Parall(*i*, *L*, *c*, *d*) for *c *∈ [-*i*, *n *- *i *- *d *+ 1].

Thus, in order to quickly verify which parallelograms are fine, we associate a *q*-hit counter to every parallelogram. First, counters are initialised for the position zero of the sliding window (lines 1 and 1). This initialisation is straightforward: for all *q*-grams occurring in [0, *L *- *q*], we check whether they create at least one *q*-hit in each parallelogram. If this is the case, the corresponding parallelogram counters are increased by one. Once the window is slided from position *i *- 1 to position *i*, the *q*-hits involving the *q*-gram that occurs at position *i *- 1 are not considered anymore, while those involving the "new" *q*-gram at position *i *+ *L *- *q *have to be taken into account. In terms of parallelograms, this corresponds to observing that the parallelograms Parall(*i *- 1, *L*, *c*, *d*) and Parall(*i*, *L*, *c*, *d*) differ only by the pairs (*i *- 1, *j*) and (*i *+ *L *- *q*, *j*) for *j *∈ [*c*, *c *+ *d*]. Therefore, in order to obtain the number of *q*-hits in Parall(*i*, *L*, *c*, *d*), we only need to subtract from the number of *q*-hits in Parall(*i *- 1, *L*, *c*, *d*), the number of *q*-hits of the form (*i *- 1, *j*) for *j *∈ [*c*, *c *+ *d*], and we have to add the number of *q*-hits of the form (*i *+ *L *- *q*, *j*) for j ∈ [*c*, *c *+ *d*]. Thus, when sliding the window in *s *from position *i *- 1 to *i*, we just have to consider all occurrences of the *q*-grams *s*[*i *- 1, *i *+ *q *- 2] (that are leaving) and those of *s*[*i *+ *L *- *q*, *i *+ *L *- 1] (that are entering) and do the following (lines 1 and 1 of algorithm 1). For any occurrence *j *of the entering *q*-gram, we have a *q*-hit (*i *+ *L *- *q*, *j*) and we increment the counters (line 1) of all parallelograms to which this *q*-hit belongs to. Conversely, for any occurrence *j *of the leaving *q*-gram, we have a *q*-hit (*i *- 1, *j*) that no longer involves the word of the current sliding window, and hence we decrement the counters (line 1) of all the parallelograms it belongs.

Observe that each *q*-hit would belong to *d *+ 1 consecutive parallelograms, for example the *q*-hit (*i*, *j*) belongs to the parallelograms Parall(*i*, *L*, (*i *- *j*) - *k*, *d*) for *k *∈ [0, *d*]. As a result, for each *q*-hit, we should update *d *+ 1 counters. In order to reduce this number, we apply a strategy that was already used both in SWIFT[6] and in QUASAR[4]. We enlarge the parallelogram from *d *+ 1 diagonals to *d *+ *b *diagonals (SWIFT actually uses *d *+ *b *+ 1 diagonals.) where *b *≥ 1. Recall that to avoid the possible presence of two non overlapping occurrences of a repeat in the same parallelogram, we must have that the width of the parallelogram should not exceed *L*, and hence *b *must be such that *d *+ *b *<*L*.

**Figure 3:**
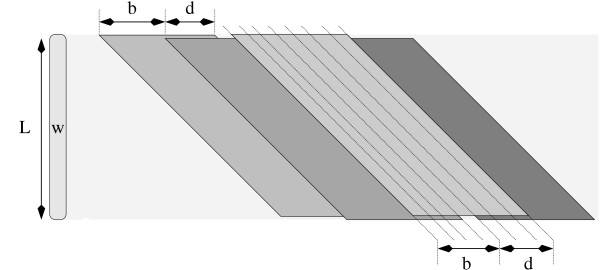
**Enlarging parallelograms from *d *+ 1 to *d *+ *b *diagonals**. Four parallelograms with *b *+ *d *diagonals, starting in diagonals *kb*, for any integer *k*. Example with *d *= 3, *b *= 4. Notice that two consecutive enlarged parallelograms of this form share *d *common diagonals and that any parallelogram with *d *+ 1 diagonals is contained in one such enlarged parallelogram with *b *+ *d *diagonals.

In this way, the parallelograms Parall(*i*, *L*, *k*, *d*) for *k *∈ [*c*, *c *+ *b *- 1] are joined in the *enlarged *parallelogram Parall(*i*, *L*, *c*, *d *+ *b *- 1). In practice, this means setting a unique counter for all Parall(*i*, *L*, *k*, *d*) with *k *∈ [*c*, *c *+ *b *- 1]. Therefore, in order to search for repeats in the whole input sequence, instead of considering all Parall(*i*, *L*, *c*, *d*) for *c *∈ [-*i*, *n *- *i *- *d *+ 1], it is enough to check for Parall(*i*, *L*, *c*, *d *+ *b *- 1) with *c *= *k'b*, for $k^{\prime }\in [-\left\lfloor \frac{i}{b}\right\rfloor ,\left\lceil \frac{n-i-(d+1)}{b}\right\rceil ]$, because every parallelogram Parall(*i*, *L*, *c*, *d*) is contained in one of these enlarged parallelograms (see Figure 3 for an example). The reason for this modification is that now a *q*-hit can only be shared by $\left\lceil \frac{d+b}{b}\right\rceil$ parallelograms. Thus, from *d *+ 1 updates per *q*-hit, we reduce to $\left\lceil \frac{d+b}{b}\right\rceil$ updates per *q*-hit. This means 2 updates per *q*-hit if as default value. For *b *we adopt the smallest power of 2 (speeding up the divisions by *b*) greater than *d*, like in [6]. Since *b *parallelograms will be combined into one enlarged parallelogram, the probability that one enlarged parallelogram is judged to be fine/good/excellent increases. As concerns the filtering conditions, we just replace parallelograms that deal with *d *+ 1 diagonals with parallelograms that deal with *d *+ *b *diagonals. The filter remains lossless, but this enlargement of the parallelograms implies that it may not be as selective as it could be. On the other hand, this enlargement makes the filter much faster.

Back to the pseudocode shown in Algorithm 1, for each value of *j*, line 1 now updates up to $\left\lceil \frac{d+b}{b}\right\rceil$ counters. When a counter reaches *p*, then the corresponding parallelogram is fine. Since we can also easily check what was the last updated counter, and since the occurrences lists are ordered, we can also only update counters that were not yet updated by the current occurrences list (line 1). This allows us to easily count first projections of *q*-hits instead of simply counting *q*-hits. Doing this, when the counter of *q*-hit projections reaches *p*, then we directly detect that the corresponding parallelogram is good (line 1).

For a given sliding window *w *= *s*[*i*, *i *+ *L *- 1], we search for excellent parallelograms (line 1) only if at least *r *good parallelograms are detected (line 1). If at least *r *excellent parallelograms are detected among the good parallelograms, all the positions [*i*, *i *+ *L *- 1] corresponding to this sliding window *w *are kept by the filter (line 1). We are now going to see how we check whether a good parallelogram is excellent.

Consider two words *w *and *w' *and also a set S of *q*-hits, relative to these words, that satisfy property (5). In order to check this property, one has to detect if at least *p q*-grams occur in the same order in *w *and *w'*. To this purpose, we consider the length of the longest common ordered subset of *q*-grams occurring both in *w *and *w'*.

In practice, we define a new alphabet ${\bar{\Sigma }}_{q}$ in such a way that every possible *q*-gram (any sequence of *q *letters in Σ) corresponds to a symbol $\bar{a}$ in ${\bar{\Sigma }}_{q}$, thus |${\bar{\Sigma }}_{q}$| = |Σ|^*q*^. Given a sequence *s *on Σ, we transform it into a sequence $\bar{s}$ on ${\bar{\Sigma }}_{q}$ replacing from left to right the letter *s*[*i*] ∈ Σ by the symbol in ${\bar{\Sigma }}_{q}$ corresponding to the *q*-gram starting at position *i *of *s*. Note that |$\bar{s}$| = |*s*| - *q *+ 1. On the ${\bar{\Sigma }}_{q}$ alphabet, the longest common ordered subset of *q*-grams occurring both in *w *and *w' *is a common subsequence of $\bar{w}$ and ${\bar{w}}^{\prime }$. In particular, if we were interested in looking for the largest set S of *q*-hits that satisfies property (5), it would be enough to compute the LCS of $\bar{w}$ and ${\bar{w}}^{\prime }$, which is a very well studied problem. Given that our third filtering condition, where we must test whether a parallelogram is excellent or not, also requires that all *q*-hits of S belong to a parallelogram, then we define the following problem:

**Definition 5 (Parallelogram *q*-hits Chaining Problem) ***Given a word w *= *s*[*a *+ *L *- 1] *and a parallelogram *Parall(*a*, *L*, *c*, *d*), *we want to exhibit a set *S*of q-hits inside this parallelogram with largest size that satisfies property (5)*.

Hence, in order to check at line 1 of Algorithm 1 if a good parallelogram is excellent, we solve the Parallelogram *q*-hits Chaining Problem for this parallelogram and check if the size of the obtained set S is at least *p*. In order to solve the Parallelogram *q*-hits Chaining Problem, many strategies could be used, ranging from a simple dynamic programming approach within the parallelogram (*strategy PDP *for Parallelogram Dynamic Programming) to a sparse dynamic programming approach that takes advantage from the fact that few *q*-hits are expected on average. For the sparse solution, we used a reimplementation of Hunt and Szymanski's [17] algorithm that was presented by Gusfield in [18] using the computation of a LIS (Longest Increasing Subsequence). In this reimplementation, for each $\bar{a}$ ∈ ${\bar{\Sigma }}_{q}$ from the first word, we should provide the occurrence list of $\bar{a}$ in the second word. In our modified algorithm, only occurrences relative to *q*-hits inside the parallelogram are considered. In order to provide this set of truncated occurrences lists, we perform *L *- *q *+ 1 binary searches in the occurrences lists. We call this the *strategy PHS *(for Parallelogram Hunt Szymanski). As an alternative, a chaining [18] algorithm applied to the set of *q*-hits inside the parallelogram could also be used, but no theoretical improvement could be expected against strategy PHS.

Moreover, we have designed an optimisation that uses some simple incremental information from the test for the sliding window *w *= *s*[*i*, *i *+ *L *- 1] in order to possibly avoid such test for the next sliding windows. This works as follows. Consider a parallelogram P for the sliding window *w *= *s*[*a *- 1, *a *+ *L *- 2] for which the solution of the Parallelogram *q*-hits Chaining Problem resulted in a set of ℓ *q*-hits. After sliding the window from *w *to *w' *= *s*[*a*, *a *+ *L *- 1], and sliding P consequently, solving the Parallelogram *q*-hits Chaining Problem results in either ℓ - 1, or ℓ, or ℓ + 1 chaining *q*-hits. Hence, only parallelograms P whose value for ℓ was equal to either *p *- 1, *p *or *p *+ 1 for position *a *- 1 (word *w*) have a chance to become or stay excellent parallelograms for position *a *(word *w'*) as well. This motivates the following optimisation: we address the Parallelogram *q*-hits Chaining Problem only for parallelograms whose previous solution for it was *p *- 1, *p *or *p *+ 1. Done on top of *strategy *PHS, this is what we call *strategy PQCP *because it is our actual solution for the Parallelogram *q*-hits Chaining Problem as we motivate later with experimental results.

In order to check for non-overlapping repeats only, given a set of good/excellent parallelograms, both at lines 1 and 1, we look for a subset of non-overlapping parallelograms with maximal cardinality. This can easily be done by applying the following greedy strategy to the sequence of parallelograms ordered by increasing starting diagonals. Let Parall(*a*, *L*, *c*, *d *+ *b *- 1) and Parall(*a*, *L*, *c'*, *d *+ *b *- 1) be two consecutive parallelograms, in this order, each one with *d *+ *b *diagonals, and let Parall(*a*, *L*, *c"*, *d *+ *b *- 1) be the next one. If the first two parallelograms overlap (which means that *c' *- *c *<*L *- (*d *+ *b *- 1)), then we can remove the second one from the sequence and repeat the process for the two consecutive parallelograms Parall(*a*, *L*, *c*, *d *+ *b *- 1) and Parall(*a*, *L*, *c"*, *d *+ *b *- 1) in the remaining sequence. If they do not overlap, then we can keep the first one in the set and repeat the process with the next two consecutive parallelograms in the sequence: Parall(*a*, *L*, *c'*, *d *+ *b *- 1) and Parall(*a*, *L*, *c"*, *d *+ *b *- 1). This procedure runs in linear time. As mentioned in the introduction, and as the names suggest, the version of TUIUIU that checks for fine parallelograms is named FINE, while the version that checks for good (resp. excellent) parallelograms is named GOOD (resp. EXCELLENT).

### Looking for repeats across multiple sequences with TUIUIU*

In this section, we describe what is done in order to look for repeats across multiple sequences, modifying TUIUIU into TUIUIU*. Consider an integer *m *≥ 2 and a set of sequences *s*_1_, *s*_2_,...,*s*_*m*_. In the set of sequences *s*_1_, s_2_,...,*s*_*m*_, an (*L*, *d*, *r*)-repeat is defined as a set of *r *≤ *m *words such that given any pair of them, their edit distance is at most *d *and they occur in distinct sequences (recall that, in this context, we use the term word for a contiguous segment of one of the sequences *s*_1_, *s*_2_,...,*s*_*m*_).

**Figure 4:**
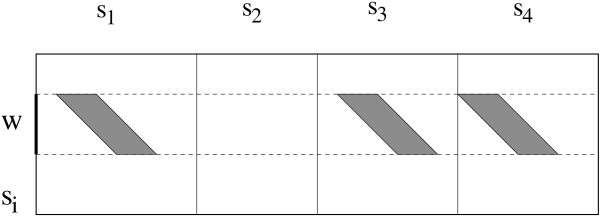
**Application to multiple sequences**. Application of TUIUIU* to multiple sequences. For a sliding window *w *on a sequence *s*_*i*_, parallelograms are tested on all other sequences. In this example, we assumed we found three fine/good/excellent parallelograms among four sequences.

Simple modifications of the algorithms FINE, GOOD and EXCELLENT are done in order to deal with these new requirements, generating the corresponding algorithms FINE*, GOOD* and EXCELLENT* that are then applied to the concatenation *s *of *s*_1_, *s*_2_,...,*s*_*m*_. While sliding the window on the word *w *along a sequence, say *s*_*i*_, we look for fine/good/excellent parallelograms in all other sequences as shown in Figure 4. If fine/good/excellent parallelograms are detected in at least *r *- 1 other sequences (different from *s*_*i*_), then we keep the word *w*. Indeed, when testing a word *w *from a sequence *s*_*i*_, we test parallelograms from sequence *s*_*j *_for *j *≠ *i*, in order to avoid to compare *s*_*i *_against itself. All counter updates are done as in TUIUIU but as soon as a desired excellent parallelogram is detected in a sequence *s*_*j*_, we skip the remaining of *s*_*j *_and go to the next sequence. Finally, if already *r *excellent parallelograms are detected, we keep *w *and try the next position. No overlap checking is done, since all obtained parallelograms detect repeated words, other than *w*, from different sequences.

### Complexity analysis

In the TUIUIU* framework, we consider *n *as the sum of the sequences length, while in the TUIUIU framework *n *is the length of this sequence. Hence, the input size is in both cases *n*. In this way, the complexity analysis is actually the same for TUIUIU and TUIUIU*.

We present a complexity analysis for EXCELLENT (which holds for EXCELLENT* too), whose pseudocode is presented in Algorithm 1. At the end, we also consider the complexity analysis of FINE (which holds also for FINE*), and of GOOD (that is the same as GOOD*). Observe that no complexity comparison is done with any other *q*-gram based filtering tool as TUIUIU is the first tool for filtering multiple repeats with edit distance.

In order to have better parameters for the complexity analysis, besides the length *n *of sequence *s*, we consider also *h *to be the number of *q*-hits and *c *the number of non-avoided computations of the Parallelogram *q*-hits Chaining Problem at line 1. For an average analysis, we consider the average number based on a random uniform distribution of *n *characters from Σ.

Concerning space usage, as described in Section "Description of the algorithm", the main data structure, the *q*-gram index built in line 1, uses an array with *n *- *q *+ 1 integers and another with |Σ|^*q *^pointers/integers. Its construction is done by applying a simple counting sort on the sequence of *q*-grams $\bar{s}$. This takes time *O*(*n *+ |Σ|^*q*^), that is *O*(*n*) if we can suppose that *q *≤ log_|Σ|_*n*. All other data structures also require *O*(*n*) memory: we have $\left\lfloor \frac{n}{b}\right\rfloor$ counters for all current parallelograms and $\left\lfloor \frac{n}{b}\right\rfloor$ skip positions (in order to avoid computations of the Parallelogram *q*-hits Chaining Problem, as we described the difference between strategies PQCP and PHS).

Concerning time, a critical parameter is the number *h *of *q*-hits. With the loops at lines 1 and 1, for each *q*-hit, we update up to $\left\lceil \frac{d+b}{b}\right\rceil$ counters at lines 1 and 1. At line 1, we execute all the *c *computations of the Parallelogram *q*-hits Chaining Problem. Using the strategy PQCP (or PHS), each Parallelogram *q*-hits Chaining Problem is solved in *O*(*L *log *k *+ *y *log *L*), where *k *is the size of the *q*-gram occurrence list and *y *is the number of *q*-hits inside the parallelogram. Therefore, each Parallelogram *q*-hits Chaining Problem is solved in *O*(*L *log *n *+ *L*(*b *+ *d*) log *L*) time in the worst case and $O(L\log\frac{n}{{\left|\Sigma \right|}^{q}}+L\log L)$ on average, assuming that the *q*-hits are sparse (as we observed in practical tests). As a result, time complexity is $O(\frac{d+b}{b}h+cL(\log n+(b+d)\log L)$ in the worst case, and $O(\frac{d+b}{b}h+cL\log\frac{nL}{{\left|\Sigma \right|}^{q}})$ on average.

Let us now estimate the values for *h *and *c*. In the worst case (*s *= *a*^*n*^), *h *= *n*^2^, but on average, *h *= *n*^2^|Σ|^-*q*^. As concerns *c*, there are at most $\left\lfloor \frac{n}{b}\right\rfloor$ good parallelograms for each position of the sliding window, thus in the worst case we have *c *= *n *$\left\lfloor \frac{n}{b}\right\rfloor$. As a first approximation, we are assuming a slightly stronger hypothesis about the sequence, a random uniform *q*-gram distribution. We can expect the probability that a parallelogram is fine (good) to be $\sum_{i=p}^{L-q}\left(\begin{array}{c} x\\ i \end{array}\right){\left({\left|\Sigma \right|}^{-q}\right)}^{i}$, where *x *= (*L *- *q *+ 1)(*d *+ *b*) is the size of a parallelogram and *p *= (*L *- *q *+ 1) - *qd*. Using Stirling's formula and the fact that for typical values *x*|Σ|^-*q*^*e *<*p*, we have

$$\sum_{i=p}^{L-q}\left(\begin{array}{c} x\\ i \end{array}\right){\left|\Sigma \right|}^{-iq}=O({\left(\frac{xe}{p{\left|\Sigma \right|}^{q}}\right)}^{p}).$$

Therefore, the expected number of fine (good) parallelograms is

$$\frac{n^{2}}{b}{\left(\frac{xe}{p{\left|\Sigma \right|}^{q}}\right)}^{p}$$

and $c=\frac{n^{2}}{b}{\left(\frac{xe}{p{\left|\Sigma \right|}^{q}}\right)}^{p}$, for any *r*.

The worst case time complexity is then

$$O\left(\frac{b+d}{b}n^{2}+\frac{n^{2}}{b}L(\log n+(b+d)\log L\right)=O\left(\frac{n^{2}}{b}L(\log n+(b+d)L)\right),$$

and the average complexity is

$$O\left(\frac{b+d}{b}n^{2}{\left|\Sigma \right|}^{-q}+\frac{n^{2}}{b}{\left(\frac{(L-q+1)(d+b)e}{p{\left|\Sigma \right|}^{q}}\right)}^{p}L\log\frac{nL}{{\left|\Sigma \right|}^{q}}\right).$$

Notice that the second part in the sum decreases as *p *increases. For *p *large enough, the average time complexity is

$$O\left(\frac{b+d}{b}n^{2}{\left|\Sigma \right|}^{-q}\right).$$

Finally, the complexity of FINE and GOOD are obtained by simply setting *c *= 0.

## Results and discussion

We now report a battery of experimental tests that were applied to TUIUIU with different filtering conditions (FINE, GOOD and EXCELLENT) and strategies for solving the Parallelogram *q*-hits Chaining Problem. As input, TUIUIU receives a sequence *s *and a set of parameters *L*, *d*, *r*, and *q*. For parameter *b*, TUIUIU takes as default value the same as adopted in [6]: the smallest power of 2 greater than *d*. Moreover, we give the user flexibility for the choice of which filtering condition to apply. As output, the user obtains a sequence where every position that does not satisfy the conditions is masked by TUIUIU. Since we do not know any other work that is a filter for multiple repeats, in particular with the same kind of output, we do not compare TUIUIU directly to other methods, but try instead to reproduce as much as possible the filtering conditions used by such approaches. In this sense, the closest method we compare TUIUIU to is SWIFT. Even though we did not report it here, we also found that the strategy of counting *q*-hits inside parallelograms used in SWIFT performs better than the strategy of counting *q*-hits inside rectangles (as is done in QUASAR[4], for instance), as it was already reported in [6]. Roughly speaking, SWIFT is a BLAST-like tool where the seeds for similarity expansions are provided not by exact matches of length W as BLAST does, but by an (*L*, *d*, 2)-repeat found by using a filter that is at the core of the SWIFT algorithm. Improving on speed seems to have been an important issue in the development of SWIFT, which also implies that we did not see reported in the paper error levels above 5%. TUIUIU can deal with bigger error levels, for instance up to 14% of the size of the repeat sought. This implies that a smaller value should be used for parameter *q*, and hence, that longer running times are unavoidable.

We start by giving a few definitions of the values that we used to evaluate the results obtained. The quality of the filtered output is measured by the ratio between the total length of the non-filtered sequence and its original length. We call this the *selectiveness *of the filter. The smaller the selectiveness, the better. On the other hand, the main resource consumed by the algoritm is the *running time *that TUIUIU takes. If we compare methods A and B, in this order, the *selectiveness improvement *(*SI*) is the quotient between the selectiveness of B and the selectiveness of A. Accordingly, we define the *speedup factor *(*SU*) of the algorithm to be the quotient between the running time spent by A and the running time spent by B. We also define the *slowdown factor *(*SD*) to be the inverse of the speedup factor. For convenience, we quite often refer to a SI/SU/SD of *x*% if the SI/SU/SD factor is 1 + *x*/100. For instance, a speedup factor 1.02 may be reported as a speedup of 2%. The experiments were run on an AMD Athlon(tm) 64 Processor 3500+ machine with 4 Gigabytes and running Linux for amd64.

### Time and selectiveness on randomly generated sequences

**Figure 5:**
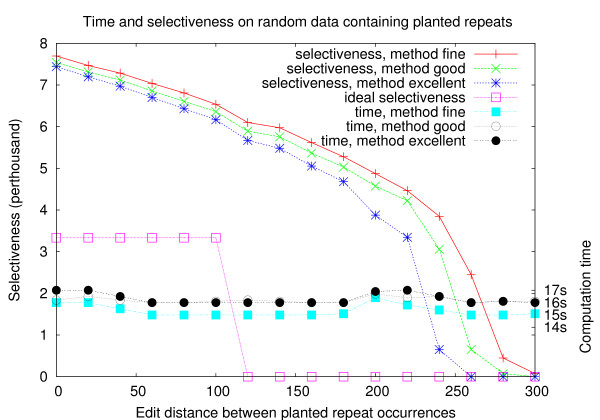
**Tests on random generated sequences**. Application of the three versions of TUIUIU with parameters (*L*, *d*, *r*) = (1000, 100, 5) on five random sequences of a total size of 1.5 Mb, each containing approximate occurrences of a planted repeat of length 1 kb. We planted repeats whose occurrences have a pairwise maximum distance that ranges from 0 to 300 (each test has the same value for all pairs). Each test was performed 20 times: the average result is reported.

We first present some tests performed on short randomly generated sequences. Each dataset is composed of five sequences of length 300 kb each, generated using a Bernoulli model (each nucleotide occurring with frequency $\frac{1}{4}$). Every sequence contains exactly one occurrence of a repetition of length 1 kb, the occurrences being distant from each other by at most *X *edit operations, with *X *ranging from 0 to 300 (that is, from 0 to 30% of the repeats length). The datasets were filtered using the methods FINE, GOOD and EXCELLENT, with parameters *L *= 1000, *d *= 100, *r *= 5 and *q *= 6 (looking for repetitions of length 1000 allowing for up to 10% differences). The results are shown in Figure 5. One can see that the computation time is only slightly influenced by the nature of the repeat, and that in this case approximately 16 seconds are required for filtering the 1.5 Mb dataset. The ideal selectiveness (in the case of absence of false positives), would be 0.33% for *X *from 0 to 100, and 0 for bigger values of *X*. As expected, one observes that the selectiveness results are better for EXCELLENT than for GOOD, which themselves are better than for FINE.

### Extensive tests with Neisseria meningitidis strain MC58

In order to compare the different variants of TUIUIU (depending on which filtering technique is used and which strategy for solving the Parallelogram *q*-hits Chaining Problem), we used a wide collection of parameter sets, applied to the DNA sequence of the *Neisseria meningitidis *strain MC58. *Neisseria *genomes are known for the abundance and diversity of their repetitive DNA in terms of size and structure [19]. The size of the repeated elements range from 10 bases to more than 2000 bases, and their number, depending on the type of the repeated element, may reach more than 200 copies. This fact explains why the *N. meningitidis MC58 *genomic sequence, with 2.3 Mb, has already been used as a test case for programs identifying repeats like in [12].

#### The best strategy for solving the Parallelogram *q*-hits Chaining Problem

In order to solve the Parallelogram *q*-hits Chaining Problem, we tested the three possible strategies described in the "Description of Algorithm 1" section. For any instance of this problem, the PDP strategy has running time proportional to the size of the parallelogram, which is (*L *- *q *+ 1) × (*d *+ *b*). On the other hand, the PHS strategy is expected to take time proportional to $\left(L\log\frac{n}{{\left|\Sigma \right|}^{q}}+y\log L\right)$, where *y *is the number of *q*-hits inside the parallelogram. Notice that *y *has a strong dependence on *q*. Increasing *q*, we decrease the probability of a *q*-hit and consequently we decrease *y*. We may expect that *y *= *O*(*L*) on average, for not too small *q*. Indeed, multiplying the expected probability Σ^-*q *^of a *q*-hit by the size (*L *- *q *+ 1)(*d *+ *b*) of a parallelogram, we may expect that *y *= *O*(Σ^-*q*^*dL*) on average, or simply *y *= *O*(*L*) for not too small values of *q*. Therefore, we expect PHS to be faster than PDP.

As concerns the PQCP strategy, it is very difficult to predict how many computations of the Parallelogram *q*-hits Chaining Problem are avoided with the optimisation it realises. The same complexity as PHS certainly holds, but we definitely expect it to be faster. The reason is that EXCELLENT parallelograms tend to be clustered together, or in other terms, parallelograms that fulfill the conditions (2), (3), (4) and (5) are usually not isolated. This happens because the probability of two adjacent parallelograms to be EXCELLENT is not independent: parallelograms close to an EXCELLENT one have an increased probability to be EXCELLENT too. To sum up, from a theoretical point of view, PQCP should be faster than PHS, which in turn should be faster than PDP.

In practice, these expectations were confirmed in the 72 tests we made with different sets of parameters on MC58. In all cases except one, the running time for the PHS strategy did not get worse in comparison to the simple PDP. In fact, the overall observed running time improvement from PDP to PHS was 1.57. In all tests, PQCP performed faster than PHS and the overall observed running time improvement from PHS to PQCP was 1.88. Hence, in all tests PQCP performed faster than the simple PDP and the overall observed running time improvement from PDP to PQCP was 3.22. Since all three strategies provide the same selectiveness, but for some cases PDP was 18 times slower than PQCP, we discarded strategies PDP and PHS from the subsequent systematic comparisons when we have to solve the Parallelogram *q*-hits Chaining Problem.

#### The FINE, GOOD, EXCELLENT variants of TUIUIU

In the Section "Methods", we saw three possible filtering conditions, depending on what kind of non-overlapping parallelograms we would like to find: fine, good, or excellent. All three filters ensure that all (*L*, *d*, *r*)-repeats are kept, so they are all lossless. In Section "Description of the algorithm", we saw the description of Algorithm 1, that implements a filter (EXCELLENT) for the third filter condition. If we remove lines from 1 to 1, we have a filter (GOOD) for the second filter condition. Moreover, if we also update all parallelogram counters (line 1) instead of only new ones as described in Section "Description of the algorithm", we have a filter (FINE) for the first filter condition. It should be said that SWIFT[6] identifies a parallelogram as a similarity region (for *r *= 2) if the parallelogram is FINE. In other words, the comparison to FINE is in some sense a comparison to the conditions applied by SWIFT for finding (*L*, *d*, *r*)-repeats for *r *= 2.

As concerns the parameter sets we used for the three algorithms when applied to MC58, we selected all combinations such that:

$$\begin{array}{lll} L & = & 50,100,200;\\ d/L & = & 4\%,10\%,12\%,14\%;\\ r & = & 5,8,13;\\ q & = & 14,13,12,11,10,9,8,7,6,5,4; \end{array}$$

and such that the restriction

*p *= (*L *- *q *+ 1) - *qd *≥ *τL*,

for *τ *= 0.08, was satisfied. This restriction was adopted because if the threshold *p *is too small, the selectiveness for any method gets bad – as one should expect – as does the running time, in particular for EXCELLENT. For instance, for *L*, *d*, *r*, *q *= 100, 14, 5, 6 (*p *= 11) we obtain a selectiveness of 99.992%, 99.957% and 99.835% for the methods FINE, GOOD and EXCELLENT, respectively. In this case, EXCELLENT is 9.36 times slower than GOOD. For this reason, in order not to spend too much running time on testing cases that would never be used anyway since the selectiveness is bad, we empirically chose a *threshold factor τ *= 0.08 for *p*/*L*. This resulted in 198 combinations for *q *ranging from 4 to 14. The combinations are split in 99 low error cases (*d*/*L *= 4%) and 99 large error cases (*d*/*L *= 10%, 12%, 14%). We are now going to comment the results reported in Table 1.

**Table 1:** FINE/GOOD/EXCELLENT systematic comparison on MC58 sequence

RESTRICTION	NB. TESTS	EXCEL. SEL.	GOOD/FINE		EXCEL./GOOD		EXCEL./FINE	
			SI	SD	SI	SD	SI	SD

overall	198	11.19	1.685	1.032	1.307	1.752	2.309	1.811

*d*/*L *≥ 10%	99	18.45	2.302	1.043	1.508	2.062	3.428	2.166
*d*/*L *= 4%	99	3.93	1.067	1.021	1.107	1.441	1.191	1.467

*q *< 7	105	14.41	2.274	1.043	1.506	1.533	3.377	1.236
*q *≥ 7	93	7.56	1.019	1.019	1.082	1.998	1.104	2.039

*p*/*L *≥ 25%	159	5.90	1.820	1.033	1.347	1.321	2.546	1.236
*p*/*L *< 25%	39	32.77	1.135	1.027	1.146	3.506	1.347	3.614

*p*/*L *> 14%	183	8.99	1.720	1.031	1.312	1.415	2.363	1.457
*p*/*L *≤ 14%	15	38.07	1.246	1.038	1.244	5.854	1.653	6.067

*L *= 200	69	6.42	1.958	1.044	1.456	1.744	2.829	1.835
*L *= 100	69	12.35	1.611	1.032	1.172	2.018	2.113	2.077
*L *= 50	60	15.35	1.454	1.019	1.292	1.454	1.938	1.479

*r *= 13	66	8.44	1.745	1.033	1.323	1.729	2.45	1.791
*r *= 8	66	11.11	1.677	1.032	1.302	1.756	2.280	1.817
*r *= 5	66	14.03	1.632	1.030	1.297	1.770	2.196	1.825

*d*/*L *≥ 10%, *p*/*L *≥ 25%	66	8.79	2.874	1.049	1.689	1.205	4.451	1.236
*q *< 7, *d*/*L *≥ 10%	78	18.42	2.631	1.047	1.626	1.710	4.038	1.800
*q *< 7, *d*/*L *≥ 25%	90	6.99	2.446	1.045	1.554	1.138	3.670	1.181

*d*/*L *≥ 10%, *p*/*L *≥ 25%, *q *< 7	63	8.78	2.962	1.050	1.722	1.188	4.614	1.237

Systematic comparison of FINE, GOOD and EXCELLENT on the MC58 data. Column *nb. tests *shows the number of set of parameters respecting the *restriction *context. Column *Excel. sel*. shows the absolute selectivity obtain thanks to method EXCELLENT. Using GOOD rather than FINE, we obtained an overall selectiveness improvement (SI) of plus 68% with a slowdown (SD) of only plus 3.2%. Using EXCELLENT rather than GOOD, for the cases with large errors (*d*/*L *≥ 10%) and not too small threshold *p *(*p*/*L *≥ 25%), the selectiveness improvement is of 69%, for a running time only 20.5% larger.

#### Variants GOOD versus FINE

We start with the comparison between FINE and GOOD. The methods are quite similar, except for an extra verification depending on whether the counter of a parallelogram to which a *q*-hit belongs was already updated or not for the current occurrences list. This extra checking introduces a small slowdown: an almost uniform slowdown of 3.2% is indeed observed in 195 out of 198 cases. In the three cases where *L *= 200, *d *= 28, *r *= 5, 8, 13, *q *= 4 – the 3 cases where the ratio between *p *and the expected number of *q*-hits in the parallelogram is smaller (85/46.2 = 1.8) – FINE was 78% slower than GOOD. These 3 degenerated cases are discarded from the subsequent running time analysis, but not discarded from the analysis of selectiveness. On the other hand, the selectiveness for GOOD is always better. Overall, in contrast to a running time slowdown of 3.2%, we observe 68.5% of selectiveness improvement on average. In Table 1, we see comparisons of GOOD against FINE restricted to the cases where certain constraints are satisfied. In this way, we can see the influence of several parameters on how much improvement we can expect from GOOD. Only in cases where *q *is bigger (*q *>= 7), no clear advantage from FINE to GOOD is observed; in fact, little difference can be observed in these cases, since the ratios are very close to 1. The advantage of GOOD over FINE is clearer for the following cases: large error rates (*d*/*L *≥ 10%), smaller *q *(*q *< 7), larger *p*, longer *L*, and larger *r*. Combinations of these restrictions improve even more the selectiveness. We have thus verified that looking for good parallelograms (as in GOOD and as TUIUIU allows the user to do) is clearly better than looking for FINE parallelograms (as FINE and SWIFT do) for smaller values of *q*, that are also required if we want to deal with larger error rates as we have done here.

#### Variants EXCELLENT versus GOOD

We now compare GOOD and EXCELLENT. If on one hand, the algorithmic differences between FINE and GOOD are quite small, the differences between GOOD and EXCELLENT are more complex mainly due to the solutions of the Parallelogram *q*-hits Chaining Problem required at line 1 of Algorithm 1. When property (5) was conceived in the design of TUIUIU, we understood that this could be a good strategy for larger rather than for smaller errors. If the parallelogram is "narrow", it is more likely that any pair of *q*-hits does already satisfy property (5). We further supposed that the extra cost of solving the Parallelogram *q*-hits Chaining Problem would be smaller for higher values of threshold *p*. What Table 1 shows us is in agreement with these expectations, since for the cases in which *d*/*L *≥ 10% and *p*/*L *≥ 25%, we obtain a selectiveness improvement of 69% in contrast to a time slowdown of 20%. Moreover, we could verify that the cases where the time slowdown is higher are those where *p*/*L *is lower. For instance, we observed that the 15 cases with time slowdown higher than 4 (ranging from 4.28 to 9.36, with average 5.85) are exactly the 15 cases where *p*/*L *≤ 14%, and we can still verify a selectiveness improvement of 55% in contrast to a time slowdown of 38% for large error cases (*d*/*L *≥ 10%) with *p*/*L *> 14% (not shown on Table 1).

On one hand, EXCELLENT always has better selectiveness than GOOD (at least equal). On the other hand, the time slowdown may not be worth it. In particular, deciding whether the time slowdown is worthwhile or not depends very much on the application the filtered sequence will be submitted to. For instance, if we have a selectiveness improvement of 30% against a slowdown of 75% (like the general average numbers for all 198 cases), it may still be worth it if the algorithm we are going to submit the filtered sequence to is, for instance, cubic, since 1.3^3 ^> 1.75. Anyway, any slowdown above 4 means that we should also consider decreasing *q *by 1, instead of changing the algorithm from GOOD to EXCELLENT, since this is the expected slowdown for this decrease. Unfortunatelly, this does not guarantee that selectiveness will improve. Moreover, inspecting the cases where *q *≥ 7 and *p*/*L *≥ 25%, one may expect the slowdown to increase if *q *also increases.

#### Variants EXCELLENT versus FINE

In order to complete these comparisons based on MC58, we proceed with the comparison between FINE and EXCELLENT. Here, like in the previous comparison, but now with even more striking numbers, it is clear that EXCELLENT performs better than FINE, bringing a selectiveness improvement of 4.45 against a time slowdown of only 25% for error cases larger than *p*/*L *≥ 25%. Overall, except for three degenerated cases where EXCELLENT got 23% faster than FINE, we obtained an average slowdown of 81% with a selectiveness improvement of 130%.

### Extra tests on Human Chromosome 22

Unfortunately, thresholds such as those present in expressions like *p*/*L *>= 0.08, 14%, 25%, *q *< 7, *d*/*L *≥ 10, depend very much on the parameters *L*, *r*, *d *and on the sequence *s *that is processed. If the sequence *s *is known to have abundant repeats, it is expected that TUIUIU will not be able to provide selectiveness better than what is imposed by the repeats present in the sequence. For instance, the human genome has a high level of ALU repeats. There is an unpublished report of a fragment of ALU Y of length 266 that repeats more than 280 times on the human cromossome 22. We decided to apply TUIUIU on this data (last assembly from University Santa Cruz, California, total length 50 Mbases, 15 Mbases of which are unknown and replaced by "N"), with parameters: *L*, *d*, *r *= 260, 13, 280 and *q *= 14, 13, 12, 11, 10, 9. Results are reported in Table 2. We observe that in all these cases GOOD is faster than FINE (24% faster on average), as in what we called degenerated cases in the MC58 analysis.

**Table 2:** FINE/GOOD/EXCELLENT comparison on Human Chromosome 22

*q*	*p*	selectiveness FINE/GOOD/EXCEL	running time FINE/GOOD/EXCEL	GOOD/FINE		EXCEL./GOOD		excel./FINE	
				SU	SI	SD	SI	SD	SI
		6.92%	501.28						
14	65	1.63%	375.59	1.335	4.253	3.198	1.056	2.396	4.490
		1.54%	1201.00+						

		6.71%	598.08						
13	79	0.88%	451.20	1.326	7.637	1.795	1.062	1.354	8.114
		0.83%	810.02						

		6.93%	761.29						
12	93	0.51%	596.23	1.277	13.653	1.289	1.075	1.009	14.684
		0.47%	768.50						

		7.24%	1067.52						
11	107	0.27%	862.29	1.238	26.321	1.099	1.136	0.887	33.684
		0.24%	947.25						

		7.88%	1647.85						
10	121	0.14%	1417.72	1.162	57.805	1.027	1.093	0.883	71.543
		0.12%	1455.64						

		8.47%	3047.66						
9	135	0.07%	2769.05	1.101	120.000	1.002	1.124	0.910	148.254
		0.06%	2773.61						

mean		2.83%	1225.10	1.240	38.278	1.568	1.091	1.240	42.550

Some tests for Human Chromosome 22 with parameters (L, d, r) = (260,13,280) and *q *ranging from 14 down to 9. The selectiveness improvement of EXCELLENT compensates for its slowdown over GOOD for *q *< 12. The selectiveness improvement of EXCELLENT or GOOD over FINE is always very large and increases as *q *decreases. Recall that SD, SU and SI stand for *speedup*, *slowdown *and *selectviness improvement *respectively. Running times are given in seconds.

Selectiveness was even better, with an average improvement of 38.3%. In these cases, we can also observe that the selectiveness improvement from EXCELLENT over GOOD, even if small, seems to compensate for the slowdown when *q *< 12, as Table 2 shows.

**Figure 6:**
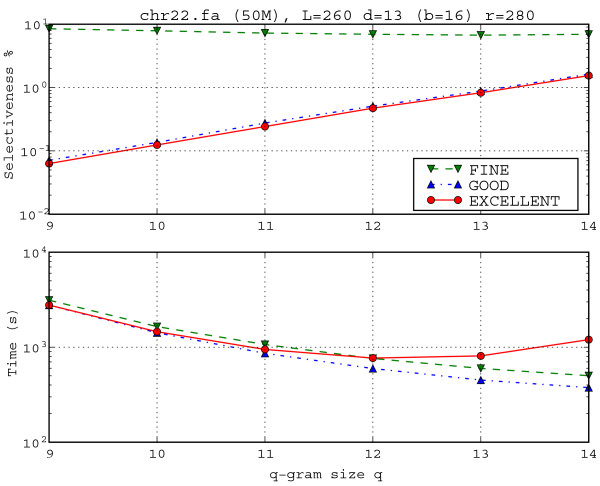
**Influence of *q*-gram size *q *over selectiveness and running time**. Influence of *q*-gram size *q *over selectiveness and running time for Human Chromosome 22 with parameters (L, d, r) = (260,13,280). Variant EXCELLENT gets slower and less selective if we increase *q *from *q *= 12 on.

Notice also that EXCELLENT always improved selectiveness over GOOD (9% on average, with a minimum of 5.5%). Moreover, the running times for EXCELLENT show that increasing *q *may not lead to a faster execution of EXCELLENT, since the fastest execution was obtained for *q *= 12. This U-type curve is illustrated by Figure 6.

**Figure 7:**
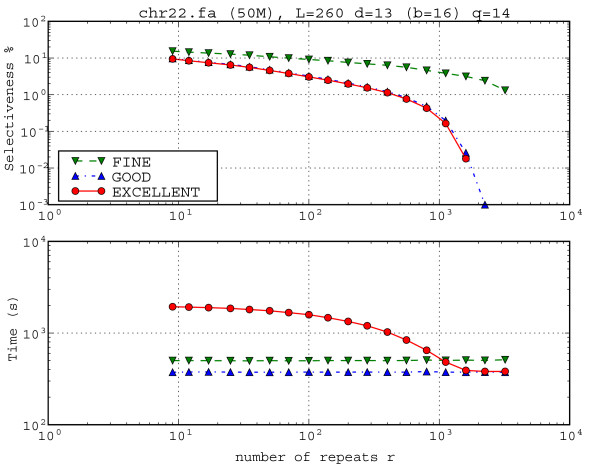
**Influence of number of repeats *r *over selectiveness and running time**. Influence of number of repeats *r *over selectiveness and running time. Human Chromosome 22 with parameters (L, d, r) = (260, 13, r) with *q *= 14. The selectiveness of 0 obtained for methods GOOD and EXCELLENT are not drawn on the log scale. As *r *grows, the selectiveness decreases because more frequent repeats are rarer than the less frequent ones. Also, variant FINE gets less selective as *r *increases. Moreover, this illustrates a case in which EXCELLENT is time consuming while not bringing an improvement on the selectiveness with respect to GOOD as we could see also in Figure 5 and Table 2.

In order to show the behaviour of TUIUIU when parameter *r *is changed, we refer to Figure 7, where *r *changes in a log scale from 9 to 3200. Notice that the running time of EXCELLENT decreases and selectiveness of all variants increases, as *r *increases. The running times of GOOD and FINE do not change. As we saw in Figure 6 and in Table 2, this parameter set with *q *= 14 is a bad choice for EXCELLENT as compared to GOOD in terms of both selectiveness and running times.

### Looking for multiple repeats across different species

In the tests described from now on, we look for multiple repeats across different species. We apply for this TUIUIU* to a dataset from orthologous regions of the cystic fibrosis transmembrane conductance regulator gene in humans (denoted by CFTR) used in [13]. From this dataset, we chose the five sequences that had no 'N': human, mouse, cow, chicken, tetra. This adds up to 5.5 Mb.

**Figure 8:**
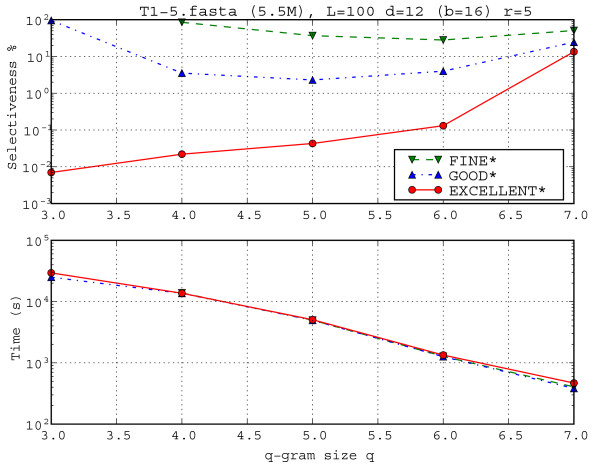
**Influence of q-gram size *q *over selectiveness and running time**. Influence of q-gram size *q *over selectiveness and running time for CFTR dataset with parameters (L, d, r) = (100, 12, 5). This test shows that EXCELLENT is essential when using a small *q*, which enables to filter for a high error rate such as 12%. For instance, with *q *= 3, EXCELLENT reduces the selectiveness of 100% observed for both FINE and GOOD to 0.01%.

**Figure 9:**
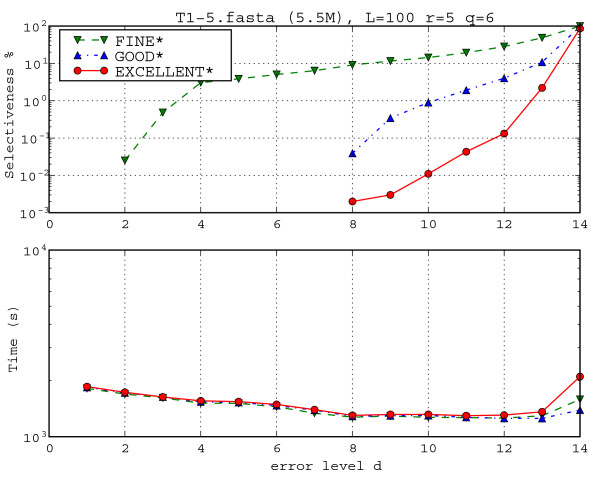
**Influence of maximal error *d *over selectiveness and running time**. Influence of maximal error *d *over selectiveness and running time for CFTR dataset with parameters (L, d, r) = (100, d, 5) with *q *= 6. A selectiveness of 0 is not drawn because of the log scale.

Like with MC58, we chose the same set of parameters, up to the fact that now we fix *L *= 100 and *r *= 5. Moreover, we added also the extreme cases where *q *= 3 and *d *= 12, 14 for the algorithms GOOD* and EXCELLENT*. In order to favour the comparisons to the MC58 cases, we discard the cases in which *q *= 3 in the average statistics. We now comment the results, shown in Table 3 and in Figures 8 and 9. position to GOOD* and EXCELLENT* that register only (*d *+ *b *- *q *+ 1).

**Table 3:** FINE/GOOD/EXCELLENT comparison on CFTR dataset

*d*	*q*	*p*	selectiveness (%)			running time(s)			Good-Fine		Excellent-Good		Excellent-Fine	
			Fine	Good	Excel	FINE	GOOD	Excel	SU	SI	SD	SI	SD	SI
	8	13	13.84	5.10	1.36	123	116	131	1.055	2.71	1.129	3.76	1.070	10.21
	7	24	12.85	1.91	0.05	371	370	385	1.003	6.71	1.041	41.90	1.037	281.28
10	6	35	14.28	0.89	0.01	1286	1292	1326	0.995	16.05	1.026	81.23	1.031	1304.19
	5	46	21.92	0.65	0.00	5080	5138	5183	0.989	33.95	1.009	235.94	1.020	8011.07
	4	57	57.96	1.02	0.00	13441	13362	13564	1.006	56.77	1.015	391.15	1.009	22208.66

	7	10	50.85	24.51	13.50	405	382	468	1.059	2.07	1.223	1.81	1.155	3.76
12	6	23	28.09	3.99	0.13	1274	1262	1338	1.009	7.04	1.060	30.37	1.051	214.04
	5	36	36.84	2.30	0.04	4972	4952	5055	1.004	16.02	1.021	53.37	1.017	855.33
	4	49	85.10	3.53	0.02	13834	13612	13676	1.016	24.08	1.004	162.60	0.988	3916.57

	6	11	99.63	96.79	85.71	1609	1405	2159	1.145	1.02	1.536	1.12	1.342	1.16
14	5	26	75.19	12.46	0.35	5017	4794	5080	1.047	6.03	1.060	35.70	1.013	215.46
	4	41	99.68	25.08	0.08	14290	13969	14112	1.023	3.97	1.010	299.49	0.988	1190.06

								mean	1.029	14.70	1.094	111.54	1.060	3184.32

Measures for FINE/GOOD/EXCELLENT on the CFTR dataset with different parameters sets (L, r = 100,5) with large errors *d *and different values of *q*. Recall that SD, SU and SI stand for *speed-up*, *slowdown *and *selectiveness improvement *respectively.

Figure 9 shows the influence of the parameter *d *on selectiveness and running time for the CFTR dataset with *L *= 100, *r *= 5 and *q *= 6. The running times of FINE*, GOOD*, and EXCELLENT* are comparable: they slightly differ only for *d *> 10. For low values of *d*, the selectiveness of GOOD* and EXCELLENT* is 0 (and indeed does not even appear in the figure because of the log scale). The reason is that the divergence of these sequences is bigger than 4%, since they belong to different species. The FINE* filter shows its limits concerning selectiveness since it does keep something (all false positives) also for these low maximal error rates.

We thus focus our attention on large maximal error rates *d*. Table 3 shows 12 parameter sets with errors *d *= 10 (*q *= 4, 5, 6, 7, 8), *d *= 12 (*q *= 4, 5, 6, 7), *d *= 14 (*q *= 4, 5, 6). In all cases we have *L *= 100 and *r *= 5. Comparing GOOD* to FINE*, GOOD* always improved the selectiveness (14.7% on average) as we can see in Table 3. Except in two cases, GOOD* was faster than FINE* (2.9% on average). Clearly on this data, GOOD* is the choice over FINE* even for larger values of *q*. Comparing EXCELLENT* to GOOD*, EXCELLENT* always improved the selectiveness (by 111.5% on average). As expected, in all cases, EXCELLENT* introduced a time overhead (9.4% on average). Only for the case *d *= 14 and *q *= 6, where the selectiveness was bad (even for EXCELLENT* with 85%), the selectiveness improvement was smaller than the observed slowdown (13% against 54%). Using the data of Table 3 in Figure 8, we can see the behaviour of algorithms FINE*, GOOD*, and EXCELLENT* when *q *changes. On one hand, EXCELLENT* always improves selectiveness as *q *is decreased – this behaviour is typical for EXCELLENT* and it is convenient since we can always improve selectiveness if we are willing to pay the extra running time associated with a decreasing of *q*. On the other hand, both FINE* and GOOD* have a U-like selectiveness curve with a minimal selectiveness (FINE* reached his minimal for *q *= 6 and GOOD* reached his minimal for *q *= 5) – this behaviour is also very typical for these methods. Since the running times of the three methods are basically comparable, due to its much better performances in terms of selectiveness, EXCELLENT* is clearly to be preferred for this data set instead of FINE* and GOOD*.

### Applying the filtered sequences to a local multiple aligner

Finally, we discuss the application of TUIUIU* as a preprocessing tool to a local multiple alignment program, using the CFTR data we described earlier. Exact local multiple aligners of *k *sequences each of length *n *take time proportional to 2^*k*-1 ^*n*^*k *^using dynamic programming. For this reason, existing multiple aligners provide only a suboptimal solution. The algorithms will still provide a suboptimal solution even when a filter is applied upstream. This is important to observe for what will follow. It means that although TUIUIU* is a lossless filter, the end result may not correspond to the optimal alignment if the aligner itself is a heuristic, or if it is designed to optimise a scoring function different from the one the filter is made for. It may even in some cases lead to a worse alignment score than the one obtained without filtering. Indeed, this is not the best use of a lossless filter, but filters, lossless or not, remain important devices for improving the efficiency and quality of multiple aligners, independent from whether the latter are exact or heuristic, as we shall see in the tests described in this section.

To our purposes, a local as opposed to a multiple aligner was also a preferable choice to illustrate the use of TUIUIU*. This limited the options, most multiple aligners being global. We decided to use GLAM2  which is an evolution of GLAM (gapless local alignment of multiple sequences, [20]) that was made for multiple alignments without gaps. Differently from its predecessor, GLAM2 allows for gaps and hence indels. Since the size of the searching space of GLAM2 is *n*^*k*^, GLAM2 samples such space using a Gibbs Sampling method for multiple alignment with simulated annealing for the optimisation step.

We first applied GLAM2 directly on the unfiltered CFTR dataset. It took 34 hours and 55 minutes to run it in order to find the best multiple alignment of the CFTR data. GLAM2 may in fact provide not just one best alignment but the ten best scoring alignments. The top scoring alignment had a score in bits of 262.977. The tests were run on an Intel(R) Core(TM)2 Duo processor with frequence of 2.40 GHz and 2 Gb of memory. We used the GLAM2 parameter that forces the alignment to involve all input sequences. Needless to say that this running time is not satisfactory.

On the same unfiltered CFTR dataset, we then applied TUIUIU*, (using the filtering conditions FINE*, GOOD* and EXCELLENT*) with *L *= 100 and *r *= 5 (the dataset contains indeed five mammalian sequences), and for *d *= 7, 12, 14, 15 (with respectively *q *= 11, 6, 5, 5). Our purpose was to then run again GLAM2 using the same parameters as above, but feeding it this time with the filtered sequences as input. In this way, we expected to reduce the searching space of GLAM2, and hence its running time. This is in fact what we can observe in the results shown in Table 4.

**Table 4:** Improvements on GLAM2 's speed and results quality after filtering by TUIUIU

			TUIUIU		GLAM2		
*q*	*d*	filter	time(s)	length	time(s)	scrbits	total time (s)
		*no filter*	*0.00*	*5518041*	*125667*	*262.977*	*125667*

		FINE*	9.26	264368	4732	244.794	4741
11	7	GOOD*	7.27	109244	2364	303.747	2371
		EXCELLENT*	**9.76**	**10256**	**228**	**283.022**	**238**

		FINE*	456.55	1556839	36357	287.102	36814
6	12	GOOD*	422.53	221127	5705	356.874	6128
		EXCELLENT*	**439.48**	**7289**	**135**	**469.664**	**575**

		FINE*	1439.12	4159686	83055	262.977	84494
5	14	GOOD*	1387.27	691442	14545	262.977	15932
		EXCELLENT*	**1499.74**	**19393**	**395**	**406.321**	**1895**

		FINE*	1640.49	5437974	107908	287.295	109548
5	15	GOOD*	1446.02	3267656	71303	256.076	72749
		EXCELLENT*	**1814.78**	**375805**	**7242**	**268.878**	**9057**

Improvements on GLAM2's speed and alignment quality after filtering the CFTR dataset with TUIUIU*. Filtration time and final length are provided for the three versions of TUIUIU* with *L *= 100 and *r *= 5. Running time, score bits of best multiple alignment found on filtered data is provided for GLAM2. Tests are done for *d *= 7, 12, 14, 15 (with respectively *q *= 11, 6, 5, 5). The last column sums the two time columns, providing the total time (filtering plus alignment).

In Table 4, we can see a first line with data involving the execution of GLAM2 on the unfiltered sequences: input length, time, and score. Then, for each pair of parameters (*q*, *d*), we show:

• For TUIUIU*, the time taken by the three versions of the filter and the length of the resulting filtered sequences.

• For GLAM2, in each case we show the time it takes to find the best alignment on the filtered data (to be consistent in the way the experiments are done and not introduce any possibly human-related bias, only the alignment at the top of the list is considered each time, as was the case for GLAM2 without filter), and the score of such alignment.

• The last column shows the sum of the time taken by the filter and that taken by the alignment on the filtered data.

It turns out that the best performances are obtained by EXCELLENT*, which lead to the fastest overall computation: the little extra time required for filtering is indeed highly compensated by the filtering power and hence by the faster alignment. Moreover, in all cases EXCELLENT* also allows GLAM2 to improve the quality of the alignment. We can explain this unpredicted behaviour by the fact that reducing the searching space allows the probabilistic searching strategy of GLAM2 to find, with much higher probability, a multiple alignment that is closer to the optimal solution.

For instance, applying EXCELLENT* with parameters (*q*, *d*) = (11, 7) decreases the running time of GLAM2 from 125667 seconds (34*h*55') to only 238 seconds (3*'*48"), that is, GLAM2 with filter runs 551 times faster than GLAM2 alone. Even adding the 10 seconds of the filtering time, we get an overall execution that is 530 times faster. Moreover, in this case the score of the best alignment also improves, going from 262.997 to 283.022. A much higher quality improvement is obtained for (*q*, *d*) = (6, 12) with a score of 469, in which case the overall time (filtering plus alignment) is also 218 times faster than without filtering (direct alignment).

In two cases ((*q*, *d*) = (11, 7) using FINE* and (*q*, *d*) = (5, 15) using GOOD*), the score obtained after filtering is smaller (the score of 262.977 became respectively 244.794 and 256.076) than the one without the filter. Besides the problem mentioned above, another reason for this behaviour is that the score function used by GLAM2 aims at optimising the score of the alignment whatever the length. The final length obtained may thus be greater than *L*. The best alignment may also contain very long gaps, and this indeed is what happens with some of the conditions and parameter sets used ((*q*, *d*) = (11, 7) using GOOD* or EXCELLENT*, and (*q*, *d*) = (6, 12) using FINE*). This is not the same objective as finding (*L*, *d*, *r*)-repeats. In the two cases were the score was smaller after filtering, the part removed by TUIUIU* apparently participated in a better local alignment *under the score function used by *GLAM2. However, our goal with these tests was to speed up the computation of the optimal alignment, and TUIUIU* clearly succeeds in that, with in most cases an improvement in the score.

As shown in Table 4, it is possible to increase *d *to 14 or even 15. In such a case, we must reduce *q *from 6 to 5 in order to keep a strong filtering condition. For *d *= 14, we can obtain an alignment with a score quite better than without the filter (406 rather than 263), with an overall execution that is 66 times faster. Finally, for *d *= 15, we can obtain an alignment with a score slightly better than without the filter, while still being 14 times faster.

## Conclusion

To the best of our knowledge, TUIUIU is the first filter for multiple repeats based on the edit distance that takes a multiple alignment task into account. Its closest ancestor, designed with a different goal, looking for (*L*, *d*, *r*)-repeats with *r *= 2 and small values for *d*, is SWIFT[6], and its filtering condition was reproduced in the variant FINE of TUIUIU. We were also able to find two improvements that led to two new filtering conditions, implemented respectively in the two variants GOOD and EXCELLENT. We tested the correctness of TUIUIU with simulated data containing planted (*L*, *d*, *r*)-repeats inserted in random data, using a very wide range of parameters sets, to check the sensibility to all parameters for all the versions. We also applied TUIUIU on three kinds of real data, the bacteria MC58, the Human Chromosome XXII, and the CFTR dataset, again using a wide range of parameter sets. GOOD was clearly better than FINE, for all kinds of data and almost all parameter sets. EXCELLENT improved considerably the selectiveness, with some overhead, and became clearly advantageous for large error rates and not too small threshold *p*. This happened in all datasets, with clearer effect on the MC58 and CFTR datasets. More tests, with wider ranges of parameter sets, are expected in a future work for Human Chromosome XXII, but the abundance of repeats with large error rates associated with the long lengths involved is certainly an intrinsic difficulty with this dataset. TUIUIU was applied as a preprocessing step of a local multiple alignment tool, leading to an an overall execution time (filter plus alignment) on average 63 and at best 530 times smaller than before (direct alignment) (from 34 hours and 55 minutes down to less than 4 minutes). Moreover, in this shorter time, the multiple alignment tool was often able to find a better scoring alignement. Indeed, the strong reduction in the searching space that was obtained due to the application of TUIUIU, and the ability of our filter to deal with large error rates, allowed the tool to perform better also at the qualitative level due to the removal of sequences that were not candidates for best local multiple alignment with bounded edit distance.

## Competing interests

The authors declare that they have no competing interests.

## Authors' contributions

MFS, NP and PP initiated the work, while all authors contributed to the conceptual and algorithmic choices. GS developed the prototype. Tests were mainly designed and performed by AP. All authors participated in the analysis of the results, and in editing the manuscript that was first drafted by PP.

## Appendix

### Appendix 1 – Algorithm: overview of TUIUIU

**Require: **sequence *s *of length *n*, parameters *L*, *d*, *r*, *q *and *b*

**Ensure: **set of positions of *s *that respect the third filtering condition

1: *p *= (*L *- *q *+ 1) - *qd*

2: Create *q*-gram index

3: Initialise with 0 all counters associated with the parallelograms

4: Initialise counter with respect to *q*-grams occurring in [0, *L *- *q*]

5: **for **every sliding window [*i*, *i *+ *L *- 1] ⊆ [0, *n *- 1] **do**

6:    **for **every occurrence *j *of *s*[*i *+ *L *- *q*, *i *+ *L *- 1] in *s ***do**

7:       Update the counters whose parallelograms the *q*-hit (*i *+ *L *- *q*, *j*) belong to

8:       **for **the updated counters that become *p ***do**

9:          Insert the parallelogram into the set of good parallelograms

10:       **end for**

11:    **end for**

12:    **for **every occurrence *j *of *s*[*i *- 1, *i *+ *q *- 2] in *s ***do**

13:       Unset the counters whose parallelograms the *q*-hit (*i *- 1, *j*) belong to

14:       **for **the updated counters that becomes *p *- 1 **do**

15:          Remove the parallelogram from the set of good parallelograms

16:       **end for**

17:    **end for**

18:    **if **number of good non-overlapping parallelograms ≥ *r *then

19:       **for **all good parallelograms P**do**

20:          Test whether P is an excellent parallelogram or not

21:       **end for**

22:       **if **number of excellent non-overlapping parallelogram ≥ *r *then

23:          Conserve positions [*i*, *i *+ *L *- 1]

24:       **end if**

25:    **end if**

26: **end for**
